# Supplementary material for: High-speed device synchronization in optical microscopy with an open-source hardware control platform
Source: Sci Rep. 2019 Aug 21;9:12188. doi: 10.1038/s41598-019-48455-z (PMC6704125; doi:10.1038/s41598-019-48455-z)
Supplement: Supplementary file 1 — Supplementary Notes and Figures [file 41598_2019_48455_MOESM1_ESM.pdf]

High-speed device synchronization in optical microscopy with an open-source hardware control platform

Supplementary text and figures

Marshall J. Colville[1*], Sangwoo Park[1], Warren R. Zipfel[1,2,5], Matthew J. Paszek[1,3,4,5]*

[1] Field of Biophysics, Cornell University, Ithaca, NY, 14853, USA

[2] Nancy E. and Peter C. Meinig School of Biomedical Engineering, Cornell University, Ithaca, NY, 14853, USA

[3] Field of Biomedical Engineering, Cornell University, Ithaca, NY, 14853, USA

[4] Robert Frederick Smith School of Chemical and Biomolecular Engineering, Cornell University, Ithaca, NY, 14853, USA

[5] Kavli Institute at Cornell for Nanoscale Science, Ithaca, NY, 14853, USA

*Correspondence to mjc449@cornell.edu and mjp31@cornell.edu

Supplementary Note 1: Hardware description

The controller electronics are divided into analog, digital I/O, in-circuit serial programming (ICSP) and microcontroller (MCU) subcircuits. Supplementary Fig. 1 provides a conceptual schematic of the analog subcircuit designs. Supplementary Fig. 7 shows the populated PCB assembly highlighting the various subcircuits in the physical layout. The complete schematics and design files are available at https://github.com/mjc449/SAIMscannerV3.git. Supplementary video 1 shows a brief demonstration of high-speed beam steering and excitation synchronization.

Waveform generators

The analog waveform outputs consist of a pair of matched subcircuits and are designed to provide accurate synchronization while still being independently controllable. To accomplish this each has independent DC references and direct digital synthesis (DDS) components. The output frequency and phase of each DDS is set by the MCU with ranges of ~0.1 to $12.5*10^6$ Hz and 0 to 2π, respectively. Because the DDS units share a 25 MHz master clock the relative frequency and phase settings are matched between the units, preventing drift. The DDS units have independent serial data lines to the MCU so that each can be programmed individually while maintaining common frame synchronization and serial clock signals. In this way the phase, frequency and waveform type for each unit can be updated simultaneously, ensuring consistent phase values and timing. The waveform output of the DDS units has a positive bias and relatively small amplitude of ~650 mV which is insufficient to directly control most peripheral devices. To precondition the DDS output the signal amplitude is increased and a bias is applied by a precision amplifier for each channel. The amplification and bias are controlled by trimming potentiometers on the circuit board. The waveform output amplitude from the controller is proportional to the conditioned waveform reference with 16-bit resolution over the

reference voltage range. The tunable amplifier circuit therefore provides a means to set a constant DC bias to the waveform reference signals as well as limit the maximum and minimum output values without decreasing the output resolution. For applications where a high-resolution DC signal is required the waveform generators can be disabled by setting the DDS units in reset (midscale output). Each of the two waveform outputs are controlled by independent dual-channel digital to analog converters (DACs). For each output's DAC one reference channel is the conditioned DDS. The second DAC channel uses a precision, trimmable 10 V reference and operates in a bipolar mode to provide a full-scale range of -10 to 10 V DC. At the output stage the two channels of each DAC are summed in a current to voltage output amplifier. The dual-channel DACs share a 16-bit parallel port on the MCU and common output register load signal. The DACs can be programmed independently or simultaneously by channels, allowing both independent and concurrent updates. Values are written to the outputs simultaneously by the common load signal, eliminating delays and ensuring output synchronization.

Bipolar analog outputs

In addition to the precision, 16-bit waveform analog outputs we have included a pair of 10-bit analog outputs with user adjustable references. Each channel has an independent 10 V reference and operates in bipolar mode. The references are connected to the DAC's reference inputs through a user-selectable jumper and trimming potentiometer. The jumper can be selected to bypass the center tap on the potentiometer to set the DAC output to a full-scale range of -10 to 10 V. Alternatively, the jumper can be set to connect the potentiometer center tap to the DAC channel's reference input. In this configuration the potentiometer forms a voltage divider, setting the reference value (V_{ref}) from ~0.1 to 10 V. The DAC output range in this mode is limited to $-V_{ref}$ to $+V_{ref}$, providing the full, 10-bit resolution across a wide spectrum of

applications. The DAC is connected to one of the MUC parallel ports to maximize update speeds.

8-channel 0-10 V analog outputs

The 8-channel voltage outputs are generated by pair of 4-channel DACs with a common 10 V reference. The DACs share a parallel data port from the MCU and data is written to each channel individually. However, both DACs are connected to a common output register load signal. Channels can be set individually, updating as soon as the new data is written, or simultaneously where the data for any number of the channels is loaded into the DACs then all outputs are set simultaneously. At the board level there is also a 10 V digital output and external power supply passthrough on the same pin header as the analog outputs. We use the 10 V digital line as a global shutter for our AOTF.

Digital I/O

We define 2 types of digital I/O for the purposes of this discussion: general purpose I/O (GPIO) and triggers. GPIO lines are those which the MCU reads based on polling the state of the pin within the thread of execution. Triggers are pins that are enabled as hardware interrupts. Interrupt events (change of pin state) trigger the MCU to halt its current thread of execution and jump to the interrupt service routine (ISR) associated with the interrupt source. When the ISR completes, the MCU continues the main thread of execution from where it left off. Our controller has 3 trigger pins that can be set programmatically to function as either triggers or GPIO and 5 additional GPIO pins. Of these pins, 4 GPIO pins and 2 triggers are +5.5 V tolerant and can operate on 3.3 V or 5 V logic without the need for logic-level conversion. There are an additional 4 digital I/O lines on an RJ45 connector. These can be set programmatically to GPIO,

triggers, or utilize the MCU serial communications hardware for linking other devices, such as a second controller or other custom peripherals using SPI or UART.

Connectors

We have avoided the use of obscure or proprietary connectors in the design of the controller (Supplementary Fig. 7). The waveform and bipolar analog outputs have SMA type connectors which can easily be adapted to BNC, SMB or many other coaxial types. The 8-channel analog outputs, digital I/O and ICSP have 0.1" pin headers at the board level for maximum versatility. For example, in our completed controller the digital I/O are broken out into 2 DB-9 connectors, 4 +5.5 V tolerant GPIO lines on one and the triggers and remaining GPIO line on another. The 8-channel analog output, 10 V digital pin and external power supply pins have been connected to a DB-25 in a pin arrangement matching our AOTF analog control connector.

Supplementary Note 2: Mechanical considerations in azimuthal scanning

Galvanometer scanning mirrors are economical and easy to integrate into optical systems such as the microscope demonstrated in this study. Commercial solutions that come packaged with appropriate driver circuitry are available from several suppliers and can even be salvaged from retired confocal and other laser scanning systems. While acousto-optic deflectors (AODs) have much faster response times, the challenge of integration of two orthogonal AODs for 2D beam scanning and higher price tag make them less attractive to most builders of circle scanning microscopes. One of the significant drawbacks of galvanometer scanning mirrors is that they are mechanical devices and as such have much slower responses to changes in position than AODs.

Furthermore, the momentum of the mirrors requires that the driver electronics can quickly supply large currents proportional to the step size of the command voltage when making a large step between points such as in a discrete scan at a TIRF radius.

Discrete scans ideally should match the number and frequency of scan points to some factor of the small-step response time (T_S), the 99% settling time, of the galvanometers. In the case of those used in our microscope this is 120 μs. To maintain the mirrors in constant motion, the time between steps along the circle must be at least $T_S/2$, or 60 μs in our case, however faster sampling will improve the quality of the waveform scanned. Ideally the update period would be $<< T_s$.

Another consideration in constructing a circle scanning microscope is the circular frequency. If the camera frame rate is on the order of the time to complete a single azimuthal scan the circular frequency must be matched to the frame rate or the benefits of circle scanning in terms of eliminating interference in the sample plane are decreased. When they are not matched for some portion of the exposure only a part of the azimuthal scan is completed and any variation in the excitation profile will be reflected in the fluorescence image. Matching the camera framerate to the scanning frequency is technically challenging. A simple solution to this problem is to maintain a scan frequency that is much greater than the minimum framerate such that the laser beam completes multiple scans in any given exposure. In this way the partial scan at the end of exposure has a negligible effect on the excitation profile.

To keep the mirrors settled on the desired waveform, the time between changes in the command signal must be held constant or fluctuations in the update rate will be reflected in the motion of the mirrors. Considering the case of a USB HID interface with the controller or driver circuitry this is difficult to achieve, as interrupt exchanges occur at a rate of 1 kHz. If the analog voltage,

as in the case of our controller, has 16-bit precision this equates to a maximum of 16 points per transfer (64 bytes per packet / (2 bytes per axis * 2 axes) at 1 kHz, or 62.5 μs per scan location. This also assumes that there are no missed transfers and that the latency in processing time of each report is less than the time between the report receipt and the previous change in the command signal. The constant timing constraint is further complicated by other tasks the controller is required to perform, and a high-priority timer interrupt is the most reliable solution to this problem. While interrupts take priority over the current computation, care must be taken in programming to ensure that the controller spends the minimum amount of time in the interrupt service routine (ISR) or other processes may incur an unacceptable delay while the ISR completes. Furthermore, a race condition exists between the priority level of scanning mirror updates and USB packet processing, as neither is more important than the other. With these constraints in place it is infeasible to construct a system wherein the controller relies on the instrument computer for continuous transfer of the scan coordinates.

To circumvent the problems associated with USB transfers the entire set of discrete scan coordinates can be preloaded into the controller before the experiment begins. In this case the controller's memory should ideally store all coordinates for the experiment, and at minimum the integer number of circles to be scanned at the reliable transfer rate. Take, for instance, a real-world scenario in which the controller can reliably receive and respond to 1 in 4 in reports, or 16 kB/s and each circle contains 32 points. In this case 2 successful transfers are required per scan radius, limiting the system to a minimum of 8 ms per exposure neglecting processing time for the new radius, system update time and settling time on the new scan waveform.

A more reliable mode of operation is to have all waveforms preprogrammed in the controller before the experiment begins. For an acquisition of 32 points per radius (as in the discrete scans

presented in the main text, which is still insufficient to keep the mirrors settled on the scan waveform as demonstrated in Supplementary Fig. 3) with 32 individual radii 4 kB of memory is occupied by the discrete scan points alone. In a microcontroller with limited memory the number of discrete scan points and number of scan radii quickly becomes the limiting factor as far as the experimental complexity that can be achieved.

In our controller the memory requirement is reduced to 4 bytes per scan radius when using the integrated waveform generators. The integrated direct digital synthesizers (DDS) run on a dedicated 25 MHz clock. Regardless of the system state the waveforms have a constant frequency and phase, the result of which is a constant circular scan of the laser independent of processor load. This also simplifies the process of settling the mirrors on the new scan radius during an update. By referencing the output DAC to the waveform input any changes are immediately reflected in the command signal sent to the mirrors. The result is a system in which the mirrors are settled on the new scan radius with a delay of T_s, which is the shortest possible for a mechanical mirror system.

Supplementary Note 3: Controller induced latency

The hardware controller introduces latency in the signal transmission between the camera and the excitation source (in our case the AOTF). This delay can be divided into 2 sources, the electronics and the firmware. In our system the AOTF global blank is an analog input of 0-10 V with 10 V being fully “open”. Even if we were to bypass the controller, additional electronics would be required to step up the 3.3 V camera logic to 10 V. Similarly, the microcontroller operates on 3.3 V logic, so we implement global blanking through a simple logic level converter

made up of a MOSFET and Zener diode voltage regulator on the PCB, eliminating the need for external electronics. This was a conscientious choice to optimize performance. The logic level converter simply follows the state of a single I/O pin and has a total turn-on/off time of less than 75 ns, which we consider to be negligible. The firmware, on the other hand, must enter the ISR associated with the camera's input before switching the global blank pin, which accounts for most of the delay introduced by the hardware controller. In Supplementary Fig. 2a the green trace tracks the controller global blanking output at the end of exposure and yellow trace is the camera fire signal. Although not explicitly stated in the text, the actual delay introduced by the controller is approximately 2.5 μs, which can be seen at the bottom of the figure in the line 1 to 4 transition time. This data was collected directly from the controller inputs and outputs, whereas the timing data in the main text reflects the actual shuttering time of the excitation laser. Connecting the camera directly to our AOTF would only improve the latency by approximately 20% at the start of exposure and 25% at the end of exposure. The remaining latency is due to the AOTF driver hardware and cannot be avoided.

With the camera connected directly to the blanking input it becomes more complicated to implement a user-selectable override. In fact, we typically leave the cameras idle or powered off when performing alignment and calibration operations at the beginning of each imaging session. We do this to avoid damaging the detectors but need to have the excitation enabled to observe the laser beam which would not be possible with the camera blanking scheme alone. Another consideration is the case of multiple independent excitation sources that are not controlled through a central device such as an AOTF or LED combiner. In such a system the camera alone could not discriminate between the excitation source required for a given frame and all the sources would need to be either on or off. With the controller described in our work the

multichannel output can be used to individually modulate each source independently for a given frame. While this would carry an additional latency of up to 50 μs, this is still much faster than what is achievable through software using USB communications.

Supplementary Note 4: Software development

Software control is a popular option for many researchers because of its ease of implementation. For many peripheral devices all that is necessary is a driver installation and/or configuring the control software. Hardware control can be significantly more difficult to accomplish. Our controller is designed to be both a development platform and a general solution for instrument control. The CAD files and bill of materials are freely available in our project repository and the populated board can be purchased fully assembled from several sources such as circuithub.com, where we have posted the necessary project files[1].

The controller firmware is available through our project repository and inexpensive programming hardware can be purchased from Microchip to flash the latest firmware into the MCU (PICkit 4, ~$48). We have also created a graphic interface that simplifies creating and executing circle scanning experiments as well as general system control (Supplementary Fig. 8). The software handles all aspects of communication and control of the hardware, including calibration, alignment and synchronization.

The features and applications of the controller presented in this work are only a subset of its capabilities and we envision the hardware platform being useful in many other applications. The success of many open-source projects is the collaborative input and development efforts of users and contributors. Through open-source development our project can benefit from the expertise

and feedback of users in fields other than our own, expanding on the capabilities and functionality of the controller.

The host software is divided into 2 layers, an application programming interface (API) which we have written in C/C++ using the HIDAPI library and compiles to a driver (.dll), and the graphical user interface written in C/C++ and Qt. Each of these layers builds on top of the other in the sense that the controller with firmware does not require the API or software, the API requires the firmware but can be integrated into user code without the rest of the software, and the software requires a controller with the firmware and driver. Finally, the driver and software have provisions for direct, packet level communication with the controller for firmware development and prototyping without needing to modify the GUI or driver code. We have adopted this model to provide a variety of entry points for working with our instrument controller. For instance a user wanting to develop a raster scan function in the firmware does not need to be familiar with HID protocol specifics or programming in Qt, while someone wanting to integrate the controller into a custom GUI in another programming language can access the controller functions as human-readable function calls in the API without needing to know the organization of data in the packets sent back and forth on the USB connection.

Supplementary Note 5: Incidence angle calibration

Both SAIM and MA-TIRF rely on accurate knowledge of the angle of incidence of the excitation beam. Calibration of the beam deflection as a function of command voltage is critical in both techniques. In the past we calibrated the system with a protractor placed on the microscope stage, measuring the beam deflection at several voltages. With the development of the graphical

interface software for our controller, we built in an image-based calibration tool for circle scanning. A piece of fluorescent acrylic is placed on the microscope stage directly above the objective and the laser is scanned in the plastic. An inexpensive webcam connected to the instrument computer captures images of the laser at various waveform output amplitudes directly through the controller software. The software then finds the edges of the scanned laser in the image and calculates the effective calibration value based on the index of refraction of the acrylic and the imaging medium. For SAIM experiments the imaging medium was assumed to have an index of refraction of 1.34. For MA-TRIF the calibration was performed with a sample index of refraction of 1.52 corresponding to the index of refraction of the glass coverslip.

The range of angles used in MA-TIRF is beyond those measurable using the laser emission from the objective. Before MA-TIRF experiments, we verified the calibration by measuring the fluorescence of an absorbed monolayer of IgG-Alexa Fluor 568 (ThermoFisher). As the incidence angle approaches the critical angle the excitation intensity at the glass to water interface increases rapidly, and past the critical angle the intensity falls off[2]. The fluorescent monolayer was imaged from sub- to super-critical angles in steps of 0.1 degrees. The critical angle was determined as the angle at which the maximum fluorescence emission was observed. The experimentally determined critical angle was then used to verify the image-based calibration.

Supplementary Note 6: SAIM analysis

The SAIM optical model is based on the fluorescence interference-contrast microscopy model and has been reported many times in the literature[3–5]. We use the simplified mathematical

representation of Carbone et. Al. in a highly optimized fitting program written in C++ with the Math Kernel Library (Intel). The program uses an unbounded trust-region solver, and as such the choice of initial parameter values can lead to erroneous outputs corresponding to the nearest local minimum of the nonlinear least squares problem rather than the true value. For the samples used in this study we set the initial value of height to the average values known from the literature[6]. We have found that the solution is relatively insensitive to the initial values of intensity (A) and background (B). However, a good approximation of intensity is 80% of the difference between the highest value and lowest value in the image series for a given pixel and background is simply the lowest value. Initially we implemented the analysis routine in a multithreaded MATLAB application, however after multiple rounds of optimization analysis of a 2048 by 2048 pixel SLB image set would take between 4 and 6 hours on a 6-core Intel Core i7 5820k processor running at 3.3 GHz. With careful optimization and the low-level memory access provided by C++ we can analyze the same dataset in < 30s. The analysis code supplied in the software repository is a simple program for analyzing a single dataset, however we have written it in such a way that the processing functions are contained in a single class for portability.

In this work we define signal to noise (S/N) as the root mean square background subtracted prediction divided by the root mean square residuals, or

$$S/N = \frac{(f(\theta_i, \beta) - B)_{rms}}{\left(I_i - f(\theta_i, \beta)\right)_{rms}}$$

Where $f(\theta_i, \beta)$ is the optical model, θ_i is the vector of incidence angles, $\beta = (A, B, H)$ is the parameter vector, B is the background, and I_i is the observed intensity in frame i. Additionally

we have found the combined evaluation of R^2 and S/N for a given pixel a better metric of the quality of the fit than R^2 alone.

Supplementary Note 7: Firmware architecture

The controller firmware used in this study was developed for running circle-scanning acquisition sequences, and as such has been optimized for high speed, sequence-based applications. The controller's functions in these applications can be generally divided into acquisition and non-acquisition tasks. A few examples of non-acquisition tasks are changes in the waveform frequency, phase offset, opening and closing a mechanical shutter or manually adjusting a laser intensity when setting up an experiment. Acquisition tasks are those that the controller should execute autonomously while collecting data such as changing the scan angle or switching the excitation pattern between frames.

The firmware architecture is of a mixed design that uses a combination of interrupt-triggered synchronization functions and polling for USB communication. The controller, once initialized, enters an infinite loop with only 2 functions. The first checks for a USB packet from the computer, the second resets the watchdog timer. When a packet is received it is processed based on the first byte, which is the command code, in a switch case statement. For every packet received the controller sends a response that can be checked by the computer-side software. Simple commands are echoed, while other commands that request information about the controller settings, memory and state, will have a more complex response. For the reasons explained in the main text polling for USB exchanges is not appropriate for timing or high-speed operations such as excitation synchronization or inter-exposure output changes. In these cases,

the controller uses a combination of hardware (I/O pin) and timer interrupts to transfer execution from the main control loop to an appropriate interrupt service routine (ISR).

Although they add complexity to the firmware design, interrupts are an invaluable tool in achieving the level of precision control required in high-speed acquisitions. For a schematic representation of the control flow see Supplementary Fig. 10. To summarize, in a polling-based approach the controller continually executes a single, serial thread. At one or more points within this thread it evaluates the system state, including changes on input pins, elapsed time since an event occurred, or the result of incrementing some loop counter. Each of these must be checked in series which, for many checks, can add up to a significant amount of elapsed time to complete. Furthermore, at some point in the loop the controller must perform any action required as a result of these checks before the loop can complete and the checks be performed again. One of the most significant drawbacks to this approach is the indeterminacy in time to execute the control loop. If no changes have occurred the loop may complete in just a few processor cycles, while if multiple actions are required the loop may take many hundreds or thousands of processor cycles, depending on the complexity of the functions. Interrupts, on the other hand are called by hardware independent of the event loop, essentially constantly being monitored. While there is overhead associated with entering and exiting an ISR this is generally small and predictable with good programming practices. Furthermore, interrupts in the microcontroller we have used can be assigned priority levels and nested, providing a simple mechanism for a high priority event to interrupt a low priority event which has itself interrupted the main event loop.

As an example of the interrupt system, the camera exposure ISR is enabled or disabled via a USB message from the computer. Once enabled, a state change on the trigger line will cause the ISR to execute regardless of the rest of the system. The camera exposure ISR can be disabled at

any time by sending the appropriate command to the controller. The camera exposure ISR is complex, as it not only controls the excitation blanking, but also handles progressing through acquisition sequences, including changing scan radius and excitation intensities of multiple lasers. Each of these subfunctions can be directly manipulated over the USB communications, however. For instance, a routine adjustment to the excitation when first examining a sample could proceed as:

1. Disable the camera exposure ISR by issuing the "Fire off" command (0x51)
2. Set the global blank high by issuing the "Enable excitation" command (0x40)
3. Increase the excitation intensity of laser 1 to 25% with the "Set excitation channel" command (0x42 0x01 0x00 0xFF) where the parameters 0x01 correspond to channel 1 and 0x00 0xFF the new intensity value
4. Reenable the camera exposure ISR with the "Fire on" command (0x50)

Note that it is not necessary to disable the ISR before changing the excitation intensity. Changes to the scan radius, waveform frequency, phase, output etc. can all be made in a similar fashion.

Acquisition sequences, called experiments in the firmware code, are organized in the controller memory as linked lists (Supplementary Fig. 11). Each node in the list contains pointers to a set of waveform amplitudes (named a sequence in the firmware) and an excitation setting (named a profile profile). The firmware has space statically allocated for up to 32 sequences of 128 angles each and up to 32 excitation profiles. Excitation profiles and angle sequences are sent to the controller by the instrument computer prior to building the experiment. Experiments are programmed by adding nodes to the experiment list, and up to 32 separate experiments can be stored in the controller. Nodes are dynamically allocated at the time of creation and destroyed

when popped from the list or when the experiment is deleted. The length of an experiment is then limited to the free memory in the controller.

At acquisition time the instrument computer issues the command to start the experiment. Once received, the controller sets the waveform outputs to the initial values in the first scan sequence, the excitation levels to those in the first profile, sets the global blanking output low and enables the hardware trigger interrupt. At the first rising edge from the camera's exposure signal on the trigger the global blank is raised to the high level and the interrupt is changed to detect the falling edge. When the exposure ends and the trigger detects a falling edge the global blank is set low. The controller fetches the next x- and y-waveform amplitudes from memory and writes these values to the corresponding DACs. A timer is started that blocks inputs at the trigger until a user-defined system settling time has elapsed, preventing exposure of a new frame before the system has settled. We typically set this to the galvanometer mirror settling time. If the end of a scan angle sequence has been reached the controller fetches the next excitation profile from memory and makes the appropriate updates to the output values. Finally, the trigger interrupt is reset to detect rising edges and the controller waits for the beginning of the next exposure.

1. mjc449/SSv3_1 · CircuitHub. Available at: https://circuithub.com/projects/mjc449/SSv3_1/revisions/16275. (Accessed: 14th December 2018)
2. Fu, Y. *et al.* Axial superresolution via multiangle TIRF microscopy with sequential imaging and photobleaching. *Proceedings of the National Academy of Sciences* **113**, 4368–4373 (2016).
3. Paszek, M. J. *et al.* Scanning angle interference microscopy reveals cell dynamics at the nanoscale. *Nature Methods* **9**, 825–827 (2012).
4. Liu, J. *et al.* Talin determines the nanoscale architecture of focal adhesions. *Proceedings of the National Academy of Sciences* **112**, E4864–E4873 (2015).
5. Lambacher, A. & Fromherz, P. Fluorescence interference-contrast microscopy on oxidized silicon using a monomolecular dye layer. *Applied Physics A: Materials Science & Processing* **63**, 207–217 (1996).
6. Kanchanawong, P. *et al.* Nanoscale architecture of integrin-based cell adhesions. *Nature* **468**, 580–584 (2010).

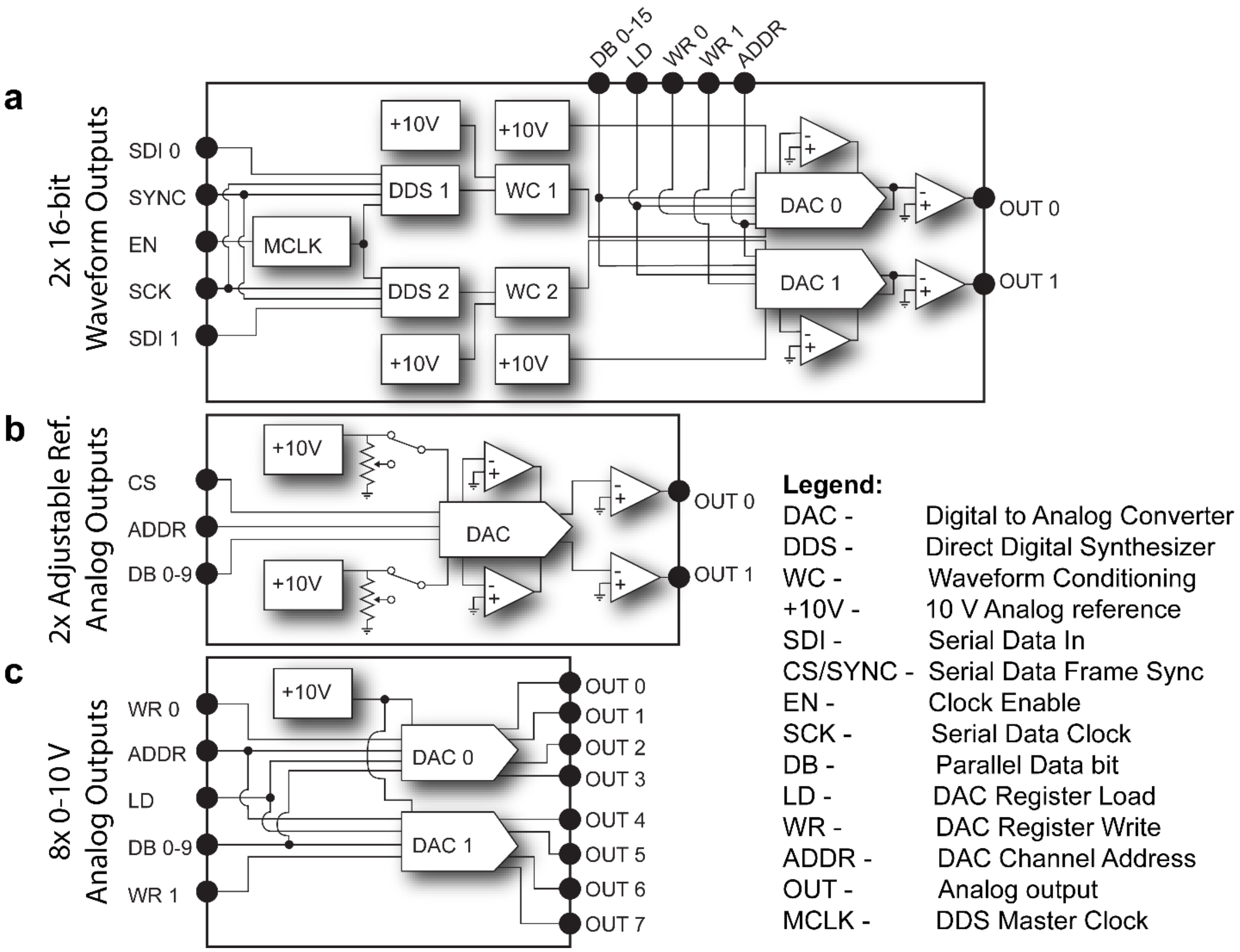

Supplementary Fig. 1: Expanded schematic diagrams of the controller analog subcircuits from Fig. 1. **a** The dual waveform generators share a master clock, sync and serial clock lines enabling simultaneous updates. The output amplitude and offset are individually adjusted by matched dual-channel DACs with a common load signal. The AC and DC outputs for each channel are summed in the final amplifier stage. **b** Auxiliary DAC outputs have individual, high precision 10 V references with selectable voltage dividers to tune the full-scale output range from ±0 to ±10 V. **c** Multichannel precision 0 to 10 V outputs are generated by a pair of matched 4 channel DACs sharing a data bus and load line. Each of the 8 outputs can be updated individually or all 8 simultaneously.

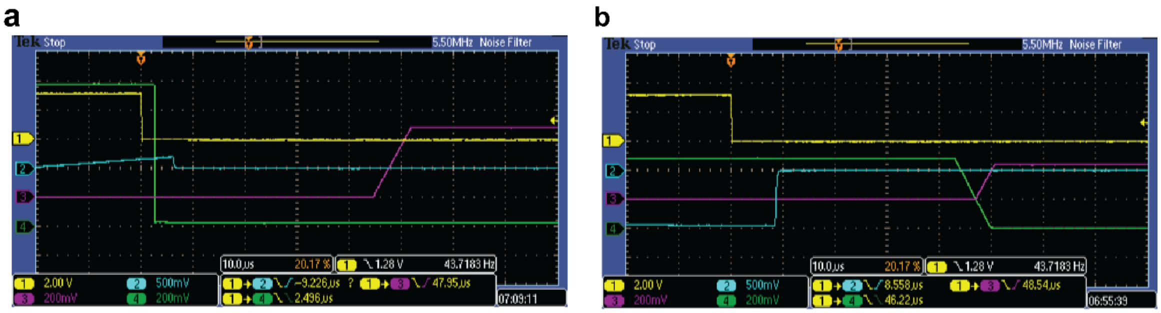

Supplementary Fig. 2: Example timing waveforms from a sequence acquisition. **a** The camera's exposure signal (Ch. 1, yellow) marks the end of the frame, triggering the controller to close the excitation shutter (Ch. 4, green). Immediately after shuttering the excitation the scanning mirror command waveform amplitude (Ch. 2, blue) is updated. Finally, the AOTF laser 0 channel (Ch. 3, pink) intensity is changed. The total update time is defined as the delay between the exposure signal trigger and the excitation amplitude settling on the new value. **b** Same as **a** with Ch4 (green) showing the AOTF laser 7 signal. All 8 channels of the AOTF are settled at the same time following the end of frame and the controller is ready to begin the next exposure in 50 µs.

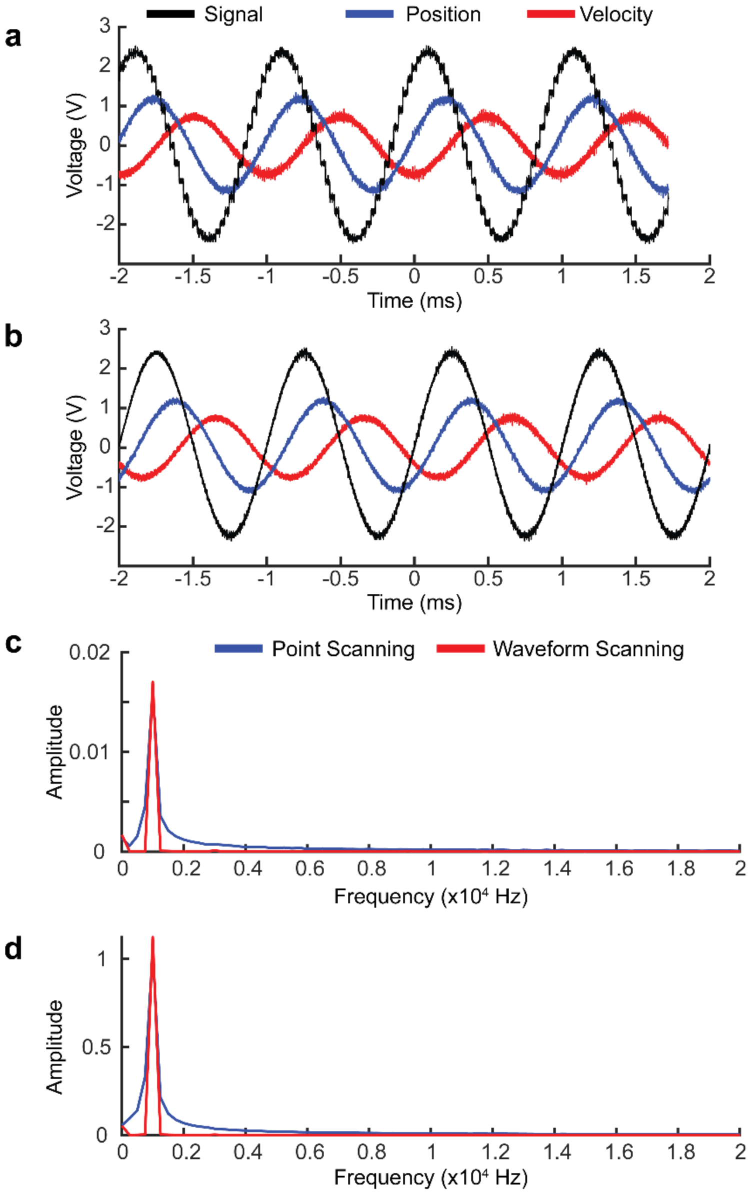

Supplementary Fig. 3: Galvanometer scanning mirror response to discretized (point scanning) and continuous waveforms in circle scanning experiments. **a** The scanning mirror is driven with a 32 point discretized sine wave at ~1 kHz (black trace) while it's position (blue trace) and velocity (red trace) are monitored. **b** Same as **a** when the command signal is replaced with a 1 kHz sine wave generated by the integrated waveform generators in the controller. Command signal amplitude in **a** and **b** is typical of a TIRF experiment. **c** Frequency spectra of the mirror position in a small angle scan of 1°. **d** Same as **c** with a large angle scan corresponding to a

typical TIRF experiment. Increased noise in the position trace from **a** compared with **b** manifests as a broadening of the frequency spectrum in **c** and **d**.

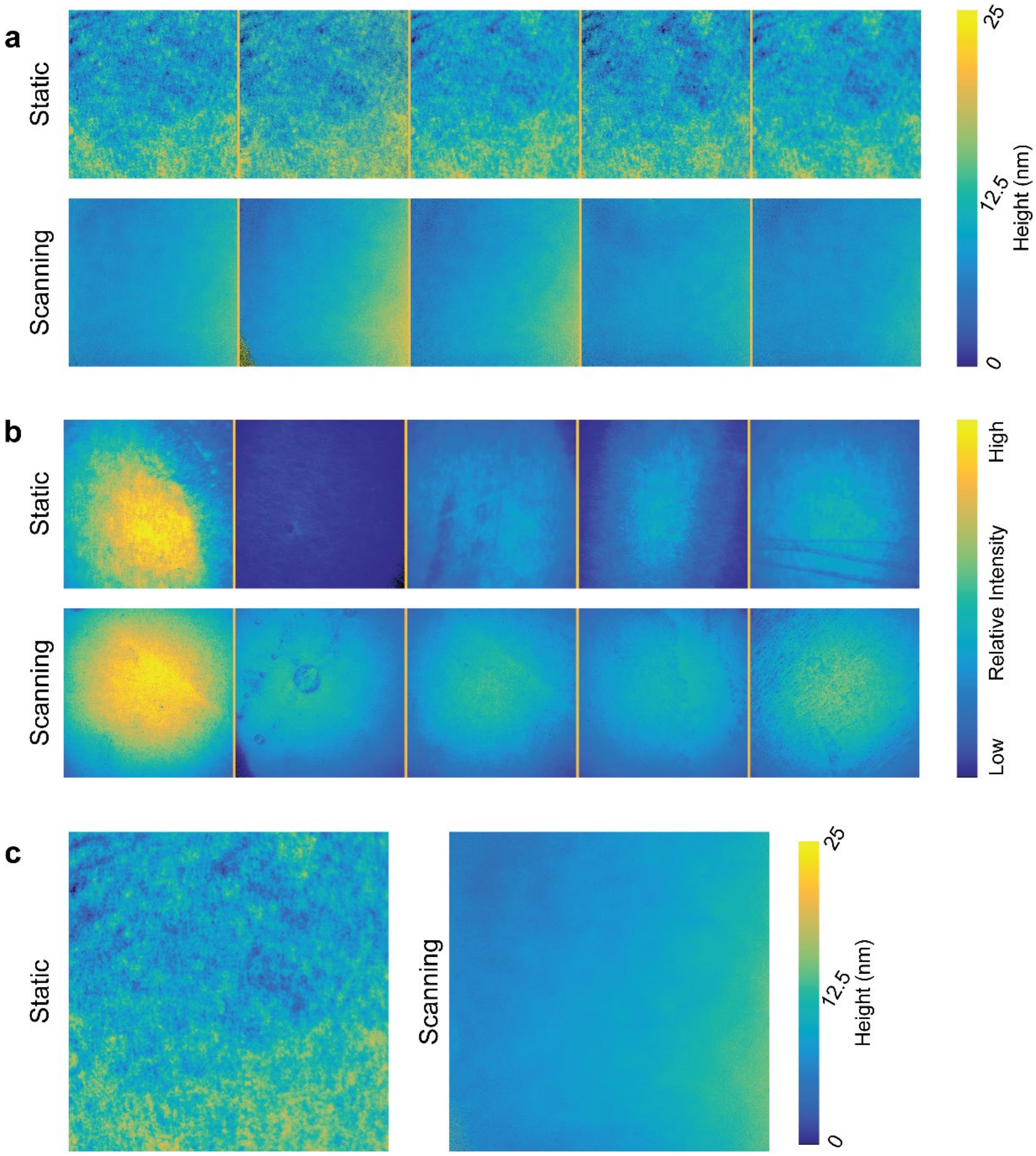

Supplementary Fig. 4: Laser speckle and fringing depend on incidence angle and lead to artifacts in SAIM reconstructions. **a** SAIM height reconstructions of 5 independent supported lipid bilayer regions acquired with static beam (top) or circle scanning (bottom) excitation schemes. **b** Relative intensity images (fit parameter A of the model function) of each bilayer region in **a**. Details in intensity images correspond to features in the bilayer resulting from

labeling variations and bilayer defects, and are similar to a widefield image. **c** Pixelwise average height of the 5 regions in **a**. Features in the average height image are the result of pixelwise incidence angle dependent excitation variations from laser speckle and fringes. All images are the full sensor area of 2048 x 2048 pixels, corresponding to a 147.9 x 147.9 μm field of view.

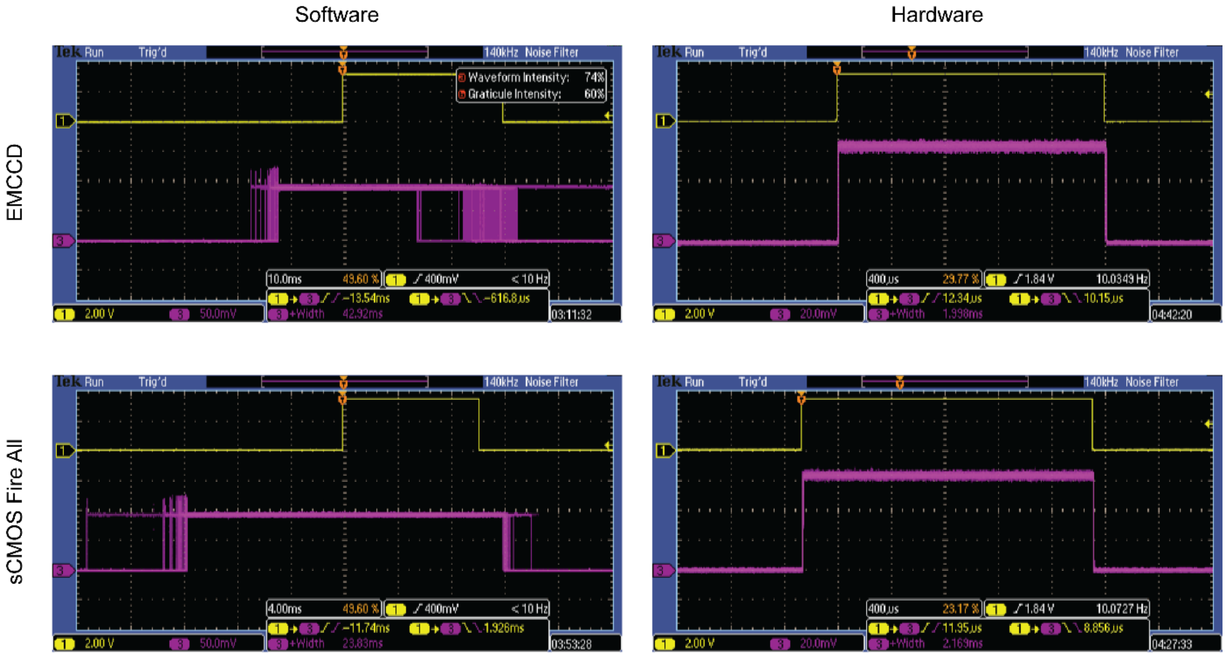

Supplementary Fig. 5: Excitation shuttering time comparison between software (left) and hardware (right) control. Ch1 (yellow) camera exposure signal; Ch3 (pink) excitation intensity collected by a photomultiplier tube placed directly in the excitation path. Traces from 100 individual exposures are overlaid to highlight the variability in excitation timing.

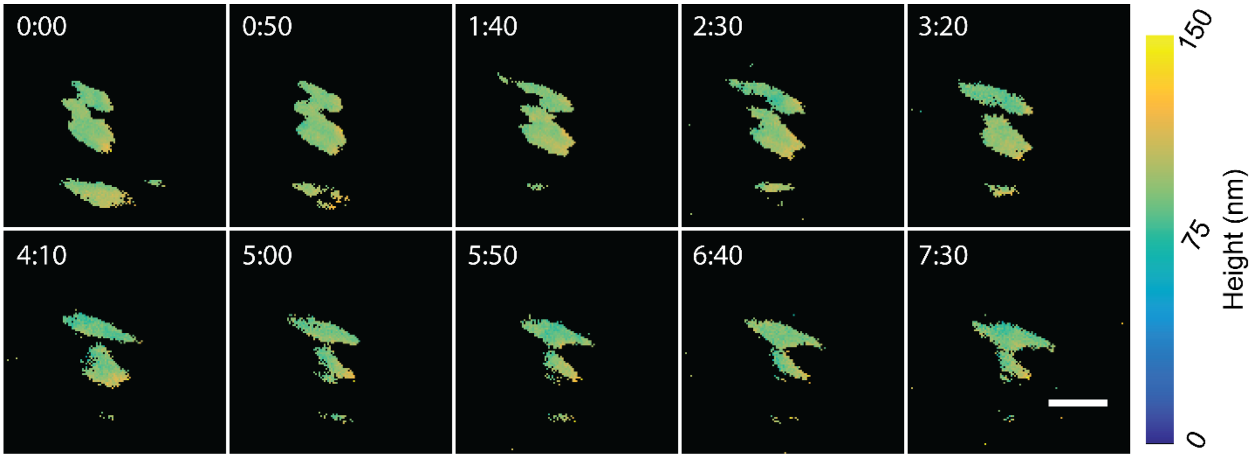

Supplementary Fig. 6: Time lapse SAIM reconstructions of the focal adhesion protein Zyxin. A live HeLa cell expressing mEmerald-Zyxin was imaged at 5 second intervals using the circle scanning excitation scheme. Scale bar 2.5 µm; time stamps are min:sec.

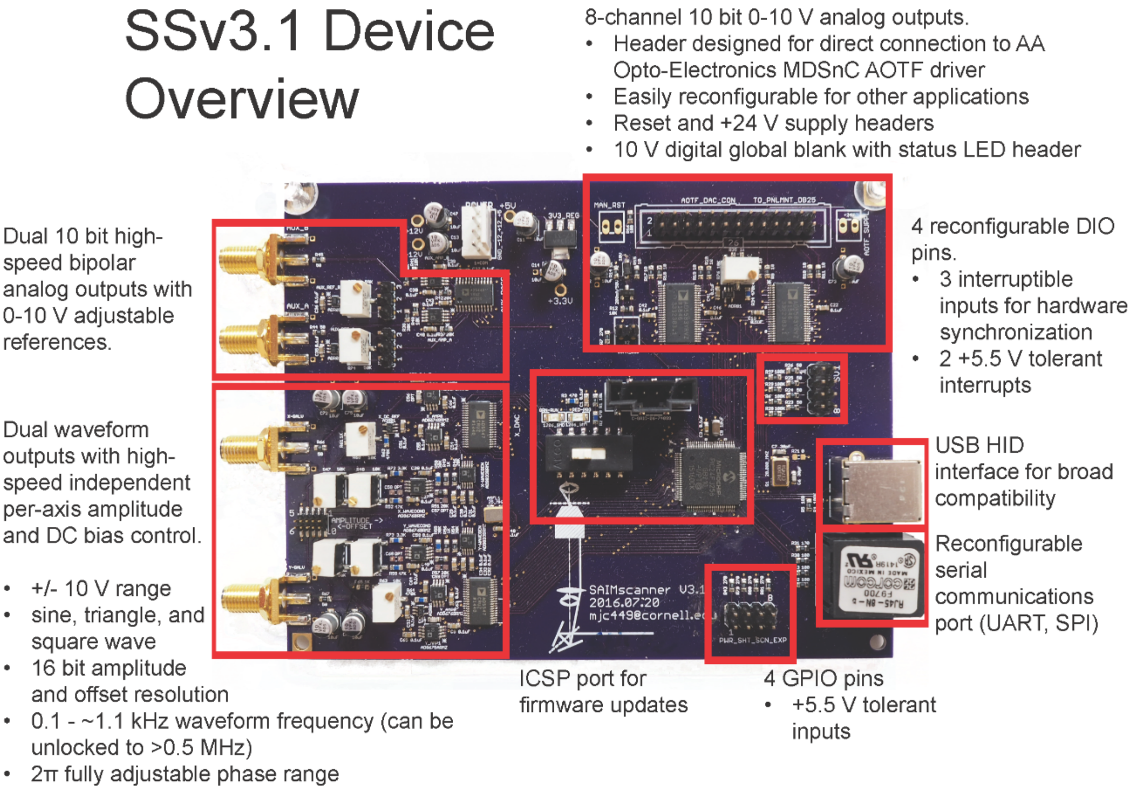

Supplementary Fig. 7: Circuit board layout. All the controller electronics are integrated into a single, compact PCB. The package sizes were chosen to strike a balance between small form factor and serviceability. Hand-building or repairing the electronics is possible with a basic, manual surface-mount device rework station.

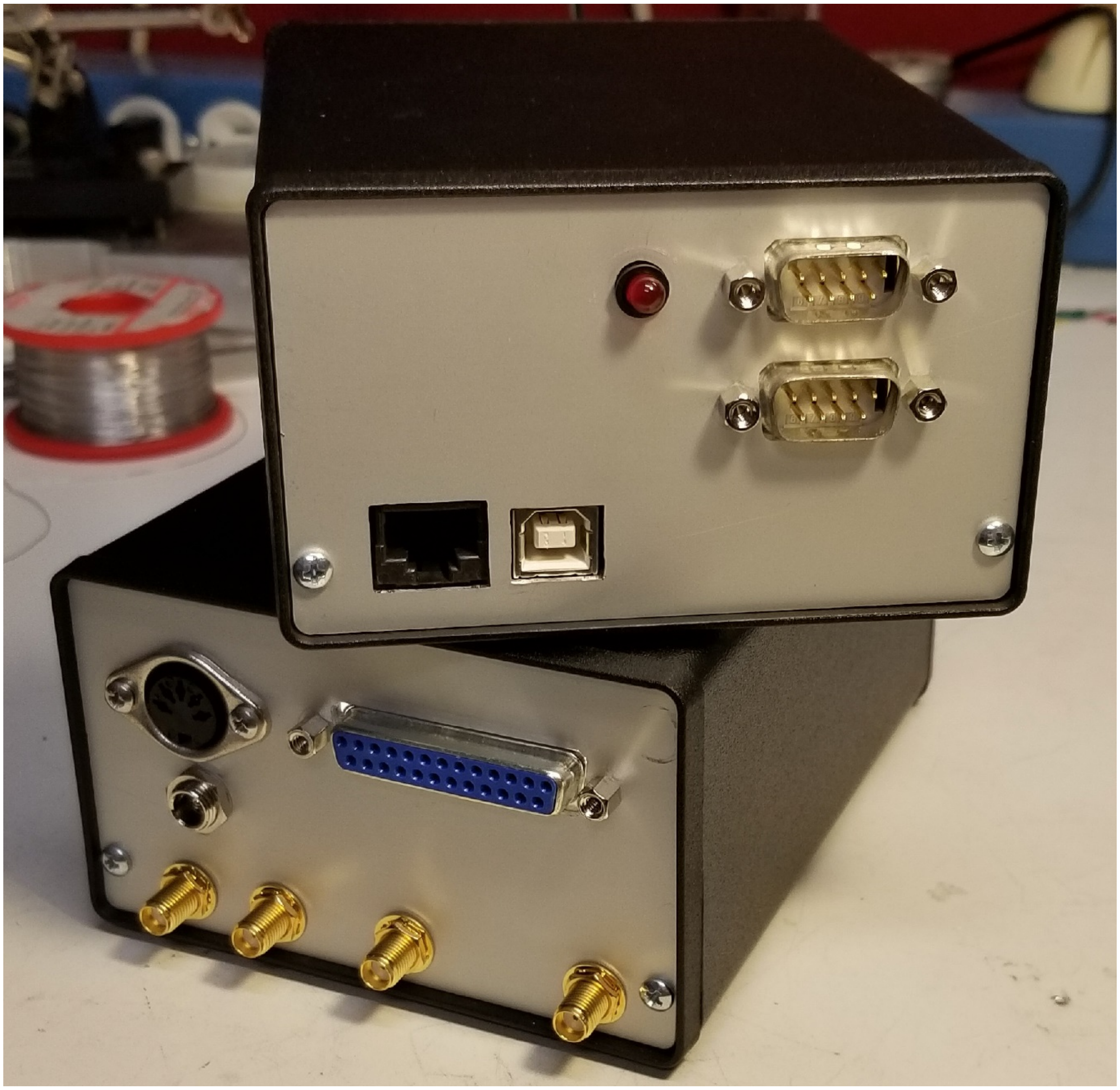

Supplementary Fig. 8: The complete controller assembly with relevant connectors.

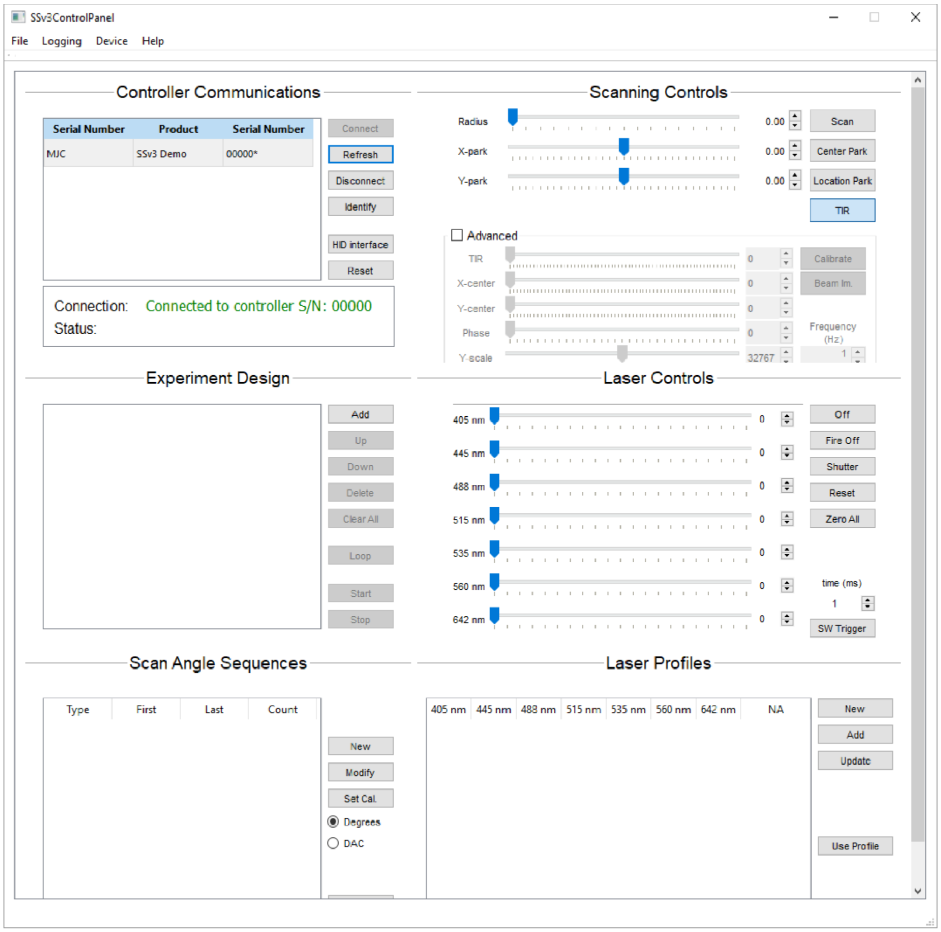

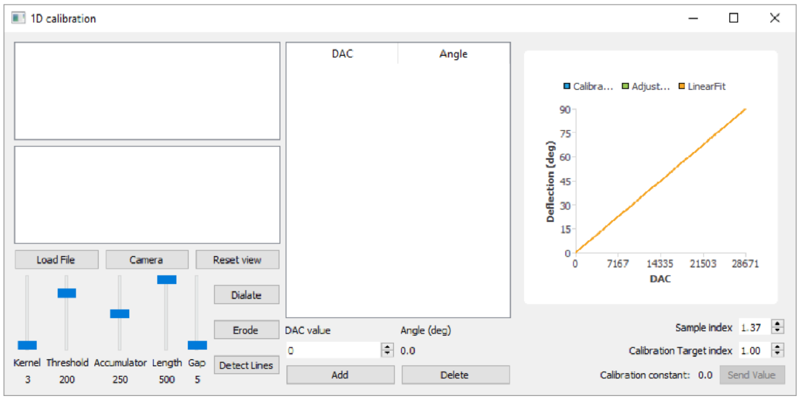

Supplementary Fig. 9: Graphical interface for circle scanning applications. Top: the main user interface can be used to create series of scan angles and sets of excitation intensities which can the be used to build complex experimental sequences. The controller can also be run manually through manipulation of the various scan and excitation controls. Bottom: Integrated program

and user interface for calibration of the excitation laser beam angle at the sample. Using a webcam pictures of the scanned beam on a vertical surface (i.e. a piece of fluorescent plastic) are taken at various scan amplitudes. The software locates the edges of the scan projection in each image and calculates the calibration constant.

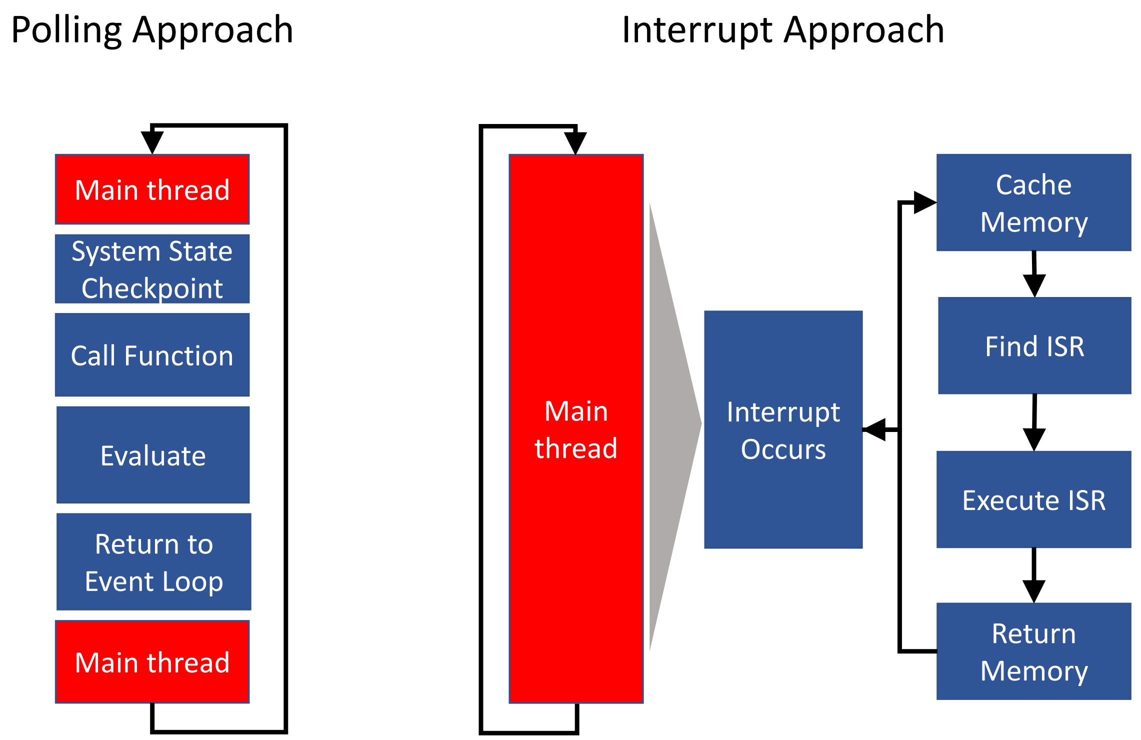

Supplementary Fig. 10: Control flow block diagrams for polling and interrupt approaches to firmware design. Left: In a polling-based approach the execution runs in a single, sequential thread. Within the thread the state of the system, including hardware I/O and program variables, is periodically sampled at pre-determined checkpoints. Right: In an interrupt-based approach the system performs low-priority tasks in the main thread of execution. When an interrupt is triggered by hardware the main thread is halted and execution is transferred to the interrupt service routine (ISR). Control is returned after the ISR completes to the main thread at the point the interrupt occurred.

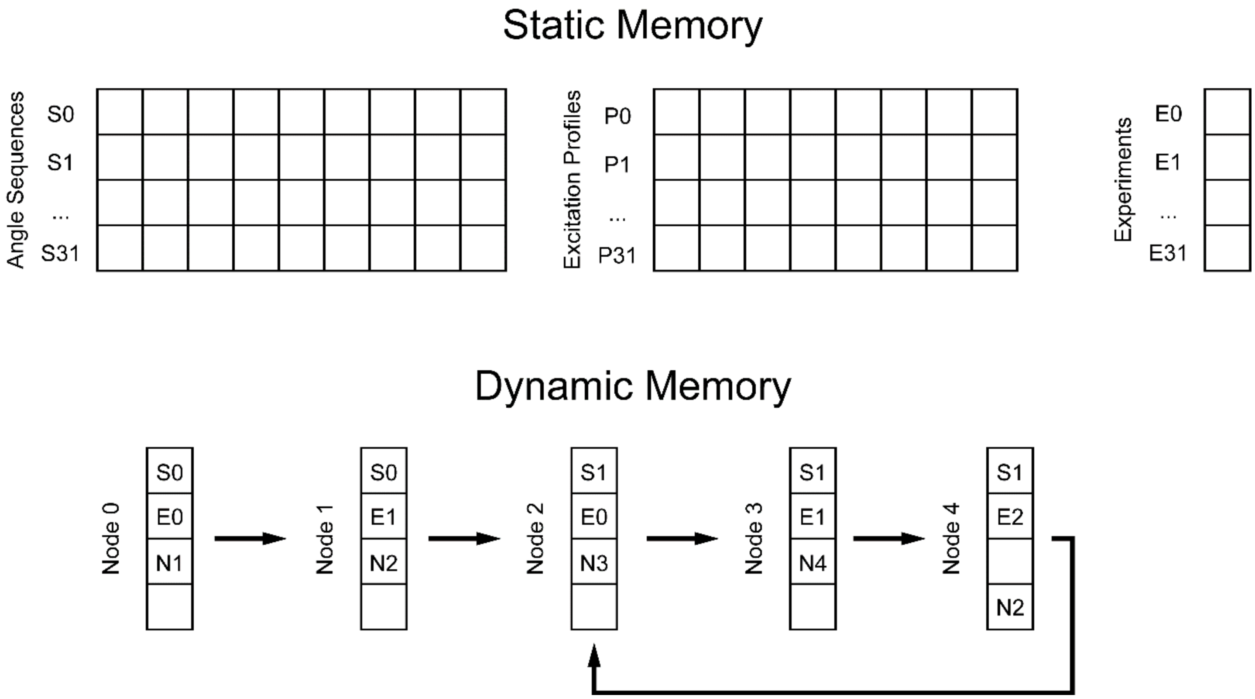

Supplementary Fig. 11: The firmware memory organization uses static storage for the experimental parameters and dynamic storage for experiment design. Each angle sequence is stored as a 1-dimensional array of up to 128 independent values. Excitation profiles are stored as 1-dimensional arrays of the 8 channel values. Experiments are created in dynamic memory as linked lists of nodes, and the address of the first node is stored in a static array of experiments. Nodes within each experiment list contain pointers to the beginning of the angle sequence, excitation profile to be used, and the next node in the list. The final node contains a pointer to the node from which to continue the experiment, allowing steps at the beginning of the experiment to be skipped. The re-use of sequences and profiles greatly reduces the memory requirements for complex experiments.

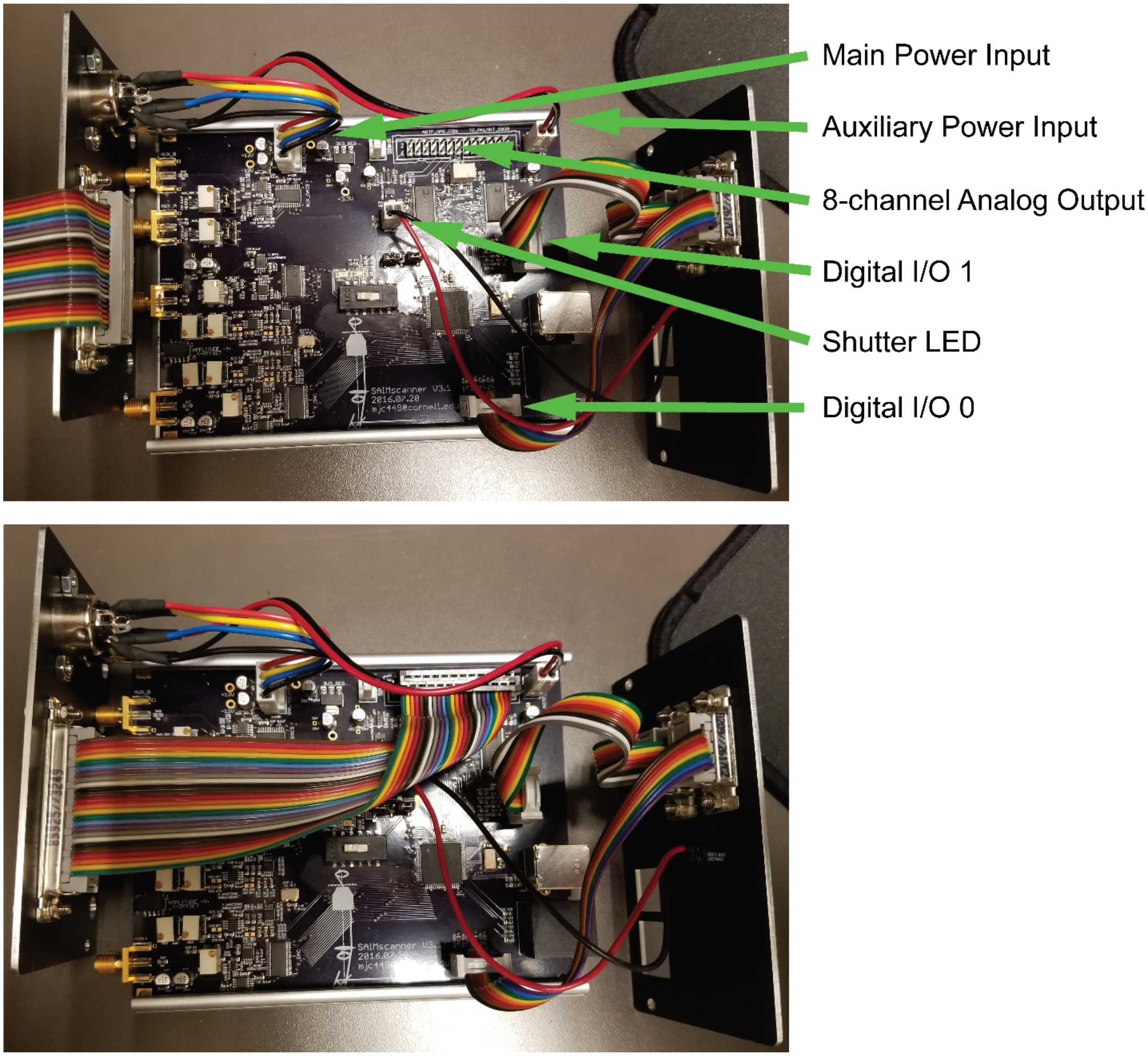

Supplementary Fig. 12: Internal connections in the completed controller assembly. Top: image of the controller enclosure with the cover removed and internal connectors labeled. The 8-channel analog output ribbon cable has been removed for clarity. Bottom: same view with the 8-channel analog output ribbon cable installed.
